# Clinicians’ views on improving inter-organizational care transitions

**DOI:** 10.1186/1472-6963-13-289

**Published:** 2013-07-30

**Authors:** Lianne Jeffs, Renee F Lyons, Jane Merkley, Chaim M Bell

**Affiliations:** 1 Keenan Research Centre, Li Ka Shing Knowledge Institute, St. Michael’s Hospital, Room 720, 30 Bond Street, Toronto, ON M5B 1W8, Canada; 2 Lawrence S. Bloomberg Faculty of Nursing, University of Toronto, Toronto, Canada; 3 Bridgepoint Collaboratory for Research and Innovation, Bridgepoint Active Healthcare, 14 St. Matthews Road, Toronto, ON M4M 2B5, Canada; 4 Dalla Lana School of Public Health and Institute of Health Policy Management and Evaluation, University of Toronto, Toronto, Canada; 5 Executive Administration, Bridgepoint Active Healthcare, 14 St. Matthews Road, Toronto, ON M4M 2B5, Canada; 6 Mount Sinai Hospital, 600 University Ave, Toronto, ON M5G 1X5, Canada; 7 Department of Medicine and Institute of Health Policy Management and Evaluation, University of Toronto, Toronto, Canada

**Keywords:** Care transitions, Inter-organizational, Orthopedic patients

## Abstract

**Background:**

Patients with complex health conditions frequently require care from multiple providers and are particularly vulnerable to poorly executed transitions from one healthcare setting to another. Poorly executed care transitions can result in negative patient outcomes (e.g. medication errors, delays in treatment) and increased healthcare spending due to re-hospitalization or emergency room visits by patients. Little is known about care transitions from acute care to complex continuing care and rehabilitation settings. Thus, a qualitative study was undertaken to explore clinicians’ perceptions of strategies aimed at improving patient care transitions from acute care hospitals to complex continuing care and rehabilitation healthcare organizations.

**Methods:**

A qualitative study using semi-structured interviews was conducted with clinicians employed at two selected healthcare facilities: an acute care hospital and a complex continuing care/rehabilitation organization, respectively. Analysis of the transcripts involved the creation of a coding schema using the content analyses outlined by Ryan and Bernard. In total, 31 interviews were conducted with clinicians at the participating study sites.

**Results:**

Three themes emerged from the data to delineate what study participants described as strategies to ensure quality inter-organizational transitions of patients transferred from acute care to the complex continuing care and rehabilitation hospital. These themes are: 1) communicating more effectively; 2) being vigilant around the patients’ readiness for transfer and care needs; and 3) documenting more accurately and completely in the patient transfer record.

**Conclusion:**

Our study provides insights from the perspectives of multiple clinicians that have important implications for health care leaders and clinicians in their efforts to enhance inter-organizational care transitions. Of particular importance is the need to have a collective and collaborative approach amongst clinicians during the inter-organizational care transition process. Study findings also suggest that the written patient transfer record needs to be augmented with a verbal report whereby the receiving clinician has an opportunity to discuss with a clinician from the acute care hospital the patient’s status on discharge and plan of care. Integral to future research efforts is designing and testing out interventions to optimize inter-organizational care transitions and feedback loops for complex medical patients.

## Background

Patients with complex health conditions require care from multiple providers and are vulnerable to poorly executed transitions from one healthcare setting to another [1-8]. Improving inter-organizational care transitions is important to address as poor care transitions can result in re-hospitalization or emergency room visits by patients, greater patient risk and increased health care spending [3-13]. Clinicians involved in care transitions may not know about the settings to which patients are transferring to or coming from [5,14-16]. Further, they may have different expertise specific to the level of complexity of the patients they are providing care to [6]. Due to this lack of knowledge, clinicians may transfer patients inappropriately or incorrectly [5,13,15]. Not surprising, many countries have national accreditation standards for organizations to ensure smooth transitions [17] and have developed financial incentives tied to readmission rates [18]. For example, the Readmission Reduction Program in the United States and being penalized financially for not meeting 30 day readmission rate targets in the United Kingdom [18].

Our current understanding of care transitions has focused on inter-shift handover (for example [19,20]); intra-hospital transfers (for example [13,21,22]); and discharge from hospital to home (for example [10,11,23-25]). Little is known about care transitions from acute care to complex medical care and rehabilitation settings and how to improve inter-organizational care transitions [13,16]. In Canada, complex continuing care settings shift the focus of priorities of patients from life and death to a focus on function and quality of life [26]. Patients transferring to this setting often have several medical morbidities coupled with mental health challenges and are socially marginalized. To address the aforementioned gap, we conducted a qualitative study that explored clinicians’ perceptions of strategies aimed at improving patient care transitions from acute care hospitals to complex continuing care and rehabilitation health-care organizations. The following research question guided our study: What strategies do clinicians recommend to mitigate safety threats and improve care transitions?

## Methods

### Setting and design

A qualitative study using individual semi-structured interviews was conducted with clinicians employed at two selected healthcare facilities: an acute care hospital and a complex continuing care/rehabilitation organization, respectively. The acute care hospital is a large, academic tertiary-care center that transfers a large volume of patients on a monthly basis. For example, on a monthly basis there are, on average, 60 orthopedic patients are transferred from the hospital to the complex continuing care and rehabilitation organization. The complex continuing care organization offers a wide range of innovative, patient-centred medical and rehabilitation services to patients living with chronic health conditions. Depending on the reason for admission, length of stay can range from four to 20 weeks. Our study included patients who had recently undergone non-elective surgery in the acute care hospital after sustaining an injury (e.g. fractured hip and extremeties). Many patients were medically complex with multiple injuries and co-morbidities and in some cases mental health issues. This study involved two orthopedic units at the acute care hospital and two units at the complex continuing care organization. Ethics approval was obtained at both participating institutions. St. Michael's Hospital Research Ethics Board approved the research study September 7, 2010 and Bridgepoint/West Park/Toronto Central Community Care Access Centre Research Ethics Board approved the research study September 23, 2010. Consent was obtained from all study participants prior to conducting the interviews.

### Selection of participants

Eligibility criteria for clinicians included that they have direct participation at the point of transition from the acute care hospital site to the complex medical care and rehabilitation organization. The recruitment process employed a combination of purposeful and snowball sampling that initially began with a broad discussion with the case managers at the acute care hospital site and the clinical managers at the complex medical care and rehabilitation organization to determine who should be interviewed [27]. This sampling approach was used to ensure that characteristics from a variety of clinical staff from the units would be captured to provide a rich, narrative account for diverse perspectives. Despite efforts to recruit physicians for interviews, no physicians from either site participated in an interview due to their lack of availability.

### Interview process

An open-ended interview guide was used to elicit the experience of clinicians around strategies to improve inter-facility care transitions. The interview questions and prompts were informed by a literature review on what is currently known about safety threats across care transitions and strategies to enhance care transitions (Table 1). This included drawing from empirical and theoretical work delineating the key factors influencing safe care transitions and strategies to enhance care transitions, mainly from discharging patients from hospital to home. To ensure clarity and relevance to the transitions from an acute care hospital to a complex continuing care/rehabilitation setting, the interview guide included open-ended questions and was assessed by the case and clinical managers. In addition, the interview guide was pilot-tested with a sample of clinicians (n = four) for clarity of the questions. A trained research staff with prior experience in qualitative methods conducted the interviews which were then transcribed.

**Table 1:** Interview guide

**Interview question**	**Prompts**
Tell me (describe for me) the strategies currently exist [at your place of work] to enhance your ability to ensure patients are safe when they transfer from acute to complex care/rehabilitation settings.	Describe the education and knowledge translation strategies around patient safety and quality.
	What ways does the organization respond to safety threats across transitions points?
Tell me (describe for me) what other strategies need to be implemented to enhance your ability to ensure patients are safe when they transfer from acute to complex care/rehabilitation settings.	What key issues exist that pose safety threats for patients transferring from acute to complex care/rehabilitation settings that care be improved?
	At a clinical level, are there changes to practice, procedures or policies that could be made to enhance your ability to ensure patients are safe when they transfer from acute to complex care/rehabilitation settings?
	At an organizational level, are there changes to practice, procedures or policies that could be made to enhance your ability to ensure patients are safe when they transfer from acute to complex care/rehabilitation settings?
	At a systems level, are there changes to practice, procedures or policies that could be made to enhance your ability to ensure patients are safe when they transfer from acute to complex care/rehabilitation settings?

### Data analysis

The analysis of the transcripts involved the content analysis approach outlined by Ryan and Bernard [28,29]. Specifically, the first step involved two research assistants reviewing the first six transcripts line-by-line independently. In this initial step, initial codes (words or sections of texts) were identified. The second step involved the two research assistants who met to discuss the codes and subsequently grouped the codes into similar content categories to form the initial coding schema. The third step involved the Principal Investigator (PI) comparing her analysis of the transcripts with the initial coding schema prepared by the research assistants. During this step, the PI was able to address any discrepancies amongst the two research assistants with codes being included in the coding schema if at least two of the three members of the research team identified the text in the transcript as a code. As a cross-checking measure, reliability amongst the research staff was determined from this third step and the remaining transcripts were analyzed to create the second coding schema. The PI then applied the coding schema to all the transcripts and revised the coding schema which was then reviewed with the research staff to achieve consensus on the final coding schema. The reporting of the study findings adhered to the RATS guidelines for reporting qualitative studies.

## Results and discussion

### Participant characteristics

In total, 31 interviews were conducted with clinicians at the participating study sites (Table 2). No demographic data other than role was collected.

**Table 2:** Participant characteristics

**Acute care hospital (n = 14)**	**Complex medical care and rehabilitation organization (N = 17)**
2 Charge nurses	4 Registered nurses
6 Registered nurses	3 Registered practical nurses
2 Physiotherapists	5 Physiotherapists
1 Physiotherapist aide	2 Occupational therapists
1 Occupational therapist aide	1 Pharmacist
1 Pharmacist	2 Dieticians
1 Dietician	

### Themes

Three themes emerged from the data to delineate what study participants described as strategies to ensure quality inter-organizational transitions of patients transferred from acute care to the complex medical care and rehabilitation hospital. These three themes are: 1) communicating more effectively; 2) being vigilant around the patients’ readiness for transfer and care needs; and 3) documenting more accurately and completely in the patient transfer record (Table 2).

### Communicating more effectively

This first theme reflected how the study participants described the need for teamwork and accountability for knowing what is going on with the patient. Having better quality of communication across multiple teams was identified as a key strategy to enhance quality inter-organizational transitions of patients at both team and inter-organizational levels.

#### Within the team

At the team level, study participants identified the need for all those involved in providing patient care to know what is going on and relay pertinent information to each other to ensure continuity of care. Other study participants identified structures that were put in place (e.g. huddles and monthly professional meetings) to ensure that patient care issues and safety threats were identified. The following narratives illustrate this sub-theme.

“Everyone should be aware from the beginning of what’s going on with the patient and all that information should be relayed to the next person who’s taking care of the patient, whether it’s a nurse, whether it’s a physiotherapist.”(Physiotherapist)

“Instead of writing it on our little transfer list, make sure you specifically talk to that person who is providing direct care to that patient to make sure that they know what the plan is. I feel that information needs to be transmitted to the people who are providing direct care.” (Nurse)

“We do wound care rounds, so the occupational therapist in collaboration with the wound care nurse specialist each week. Communication is key, the safety huddles that we are having and identifying the patients at risk for falls is huge assisting in that.” (Occupational Therapist)

#### Across organizations

At an inter-organizational level, study participants from both health-care organizations described the need for staff to have direct verbal communication with the most responsible clinician (e.g. nurse to nurse, physiotherapist to physiotherapist) prior to the transfer process and once the patient has arrived at the complex medical care and rehabilitation organization hospital. As part of this sub-theme, the following strategies were identified: 1) staff at the acute care hospital to inform the complex medical care and rehabilitation organization staff verbally that the patient is being transferred and provide information on the patient’s status, identified risks (safety threats) and care needs; 2) staff at the complex medical care and rehabilitation organization to indicate to the acute care hospital that the patient and relevant information on the patient’s status and care plan have been received; 3) have feedback loops on the quality of the transfer process and how the patient is doing; and 4) provide opportunities for the staff and physicians to gain knowledge around what and how care is provided at the different level of care organizations (acute and complex continuing care and rehabilitation in this study). This sub-theme is elucidated in the following excerpts from the interviews.

“A good verbal report from our facility to their facility would be very important to explain the little tid-bits about the patient that we’ve picked up on as we’ve had them for the last three-four days. The biggest one would be giving a telephone hand-over, transfer of the accountability.” (Nurse)

“I don’t think that’s something that’s fed back to us very often at the frontline staff level so it’s difficult for me to say what are the types of things that can or do go wrong in these transitions of care or transfers. It’s pretty hard to make a change so communication and reporting back on these issues would be helpful. More communication needs to occur to inform the frontline staff like myself of these problems so that we can also contribute to possible solutions for any safety issues that are identified.” (Physiotherapist)

“If we can get a discharge report of why things have changed, if we can get something from the middle man, I guess is what we kind of call him, if we can get that information from the pharmacist of why things have changed it makes our life a little easier to parlay that information to them.” (Pharmacist)

“We should have more information on the receiving end, we need more information from there. What do they do? How their system works? What the rules are when the patient leaves here and goes there? We need some of that information, because I don’t know how their assessment system is.” (Nurse)

### Being vigilant around patients’ readiness for transfer and care needs

This second theme reflects how both site cohorts identified there was room for improvement in being vigilant around patients’ readiness for transfer and meeting their care needs as one study participant noted “*we have a lot of work to do to get it up to the ideal*”. A key part of being vigilant at the acute care hospital was having team members as part of their discharge planning “*collectively keep an eye*” on the patient’s status and their potential for risks (e.g. falls, pressure ulcers) and communicate and consult with each other around the patients’ status, as noted below:

“We can all collectively keep an eye out and try and help these people if they are doing something unsafe, we know that they are at high risk for falls so we can go help them and get them the assistance that they need or the most appropriate person to work with them at the time. Just being mindful, and being aware of the patient, knowing your patients, and referring to the facility notes. Having that information is important and ensuring that that information is reviewed from previous chart.” (Occupational Therapy)

“I think the care that we take in communicating with our patients and our families helps tremendously to prevent a lot of difficulties. Through my everyday assessment, it’s always looking at safety so their ability to mobilize safely, any factors that might make them more susceptible to falls, looking at their skin condition so all of those things and pulmonary status, keeping an eye on that and monitoring that.” (Physiotherapy)

#### Readiness for transfer

Some participants described that there was a goal to transfer surgical orthopedic patients to a rehabilitation facility on the third day post-operatively. In some cases, staff viewed that they were trying to get everything done to ensure a safe transition but were discharging them too early. To ensure patient safety, some clinicians described that they would continue to assess the patients, particularly if they were concerned around them not being ready for transfer, and notify the doctor if they viewed the patient to be unstable. Another example provided was that the pharmacists from both health-care organizations would connect around patients who presented with a complex medication history to review the patients’ medication profiles together. These two examples of this sub-theme are described below:

“We make sure that the patient is safe before we transfer them. If we notice that there is anything that is not right, we would let the doctors know. If it’s a case where we think that the transfer shouldn’t take place, because something is not right, you tell them and then the transfer is being pushed back for a later date. We continue to assess the patient and if there is an intervention to be done, it gets done.” (Nurse)

“I do like it when the rehab facility has called me just to clarify some things that were uncertain. I’m more than willing to answer any questions they have because sometimes they go abruptly. Either they’re missing information in the chart that they just want to clear with me. I think there is a healthy relationship when there’s a liaison back with our facility.” (Pharmacist)

#### Meeting patients’ needs

The second theme also included being mindful of attending to patients’ needs, including the fundamentals of care and information needs, during care transitions. Specific strategies identified by study participants included having necessary care (e.g. medication) and treatment; nutrition (e.g. water and food) and other care essentials (e.g. wash basin and urinal) available during all aspects of the transfer, and information needs addressed. This sub-theme is illustrated with the following narrative excerpts.

“We make sure that the patient is safe enough before they’re being transferred. If a patient is complaining of anything, like chest pain or anything, of course you wouldn’t transfer them. Making sure that the room is well lit and that the call bell is within reach.” (Nurse)

“With every new patient coming in they should have water, a urinal, wash basin, all at the bedside. If physiotherapy is not right there then nursing should be able to check the notes and help the patients. One of the big things that help the patient care is the continuity of having the same people every time. If they can, just keep nursing continuity.” (Physiotherapy)

“Be mindful that the patient is going to be transferring to a new facility in which they might be waiting to receive certain things like food and medication. Be mindful of that and try to give medication at an appropriate time so that when they come, they can wait a bit without having certain meds being offered to them. I think if everybody just thought smart about all of these items, a lot of the safety issues could be minimized so just consider their needs.” (Nurse)

### Documenting more accurately and completely in the patient transfer record

The third theme that emerged was the need for clinicians to document more accurately and completely in the patient transfer record. Specific recommendations included having more details from nurses on the care needs and requirements in the transfer notes, a clear description of the patient’s cumulative patient profile (e.g. how things have changed over time), and a streamlined note from all clinicians involved in the patient’s care prior to the transfer. This is noted in the following quotes from study participants.

“There is sometimes a different diet or different texture throughout so if we could stream line that and make it consistent, then it would be much safer and less time for people fishing out information. Sometimes we end up calling the acute care site and the patient is waiting.” (Dietician)

“We need more nursing notes. According to the medical record we know everything but we want to know the details such as, the patient’s bowel movements. We want a lot of details for this patient for daily activity. If we have more details we can take care of the patient safely.” (Nurse)

“The other things that should be included in the transition would be full updated notes for the receiving therapist.” (Physiotherapist)

### Discussion and implications

Our first theme underscores the importance of improving the quality of the communications within the team and unit level and between the sending and receiving health care organization level. For both levels, study participants provided some insightful, yet simple, strategies. At the unit level, the view that all clinicians providing care to the patient should be involved and know what is going on around the patients’ status and plans for transfer is consistent with what has been reported in a recent systematic review on intra-facility handoffs [21]. Further, our finding supports the view that effective communication is more than a one-way transmission [30] and requires a multi-disciplinary approach [31]. Study participants also valued structured approaches (e.g. daily huddles) to facilitating communication and collaboration amongst clinicians at the unit level. This is consistent with an evolving literature base on standardizing care transition points by the use of multi-disciplinary rounding and other communication strategies in efforts to improve patient care transitions [7,31-34].

At an inter-organizational level, some of the clinicians in our study described the value of having a verbal report from the same group of clinicians (e.g. nurse to nurse; pharmacist to pharmacist; dietician to dietician; and physiotherapist to physiotherapist) from the sending hospital to the receiving complex medical care and rehabilitation organization hospital that is tailored to what the patient’s health status is and plan of care. From these interactions, clinicians at the complex medical care and rehabilitation organization were able to clarify and further tailor the plan of care and better able to meet patient care needs. This finding is consistent with other empirical work [26,31]. The majority of clinicians were not able to connect between the acute care hospital and complex medical care and rehabilitation organization. Both sides expressed frustration of not knowing what happens to the patient once they have been transferred or not having all the information around the patient’s status and care plan. Further, some participants shared that they had minimal understanding of the nature of the care provided at the other health care organization. Consistent with other literature [5,13-15,29], these findings warrant further attention as a lack of communication between settings can result in a variety of clinical errors (e.g. omission of newly ordered medications or administration of discontinued medications) and misinterpretation of expectations.

In addition to the potential for increased risk for errors, the study finding that clinicians at each organization were working in isolation of the other organization has also been observed with staff at hospital emergency departments and staff at nursing homes [14,15]. For example, McCloskey [14,15] used the concept of ‘mindlessness’ to describe why clinicians interacted in ways counterproductive to collaboration and quality patient care during transfers between nursing homes and emergency departments. Further, working in silos was viewed as contributing to clinicians not having a full picture of a patient’s complete pathway across the course of care [35]. As mentioned by some study participants, a key strategy to improve care transitions across the two facilities was to standardize the handoff process. There is growing evidence to support that standardized approaches to the handoff process whereby expectations for each clinical speciality are acknowledged across clinical specialities results in better and efficient care during care transitions [36,37]. Key to this work is creating an established communication channel that outlines how to identify the appropriate person and how to contact that person [36-38]. Furthermore, a variety of data and channels of communication are required for meaningful information exchange during care transitions [16,31].

Our second theme elucidates the importance of vigilance of patients’ readiness for transfer and care needs to ensure quality care transitions. As part of being vigilant in assessing patients, some clinicians described that by keeping an eye on the patient they were able to discern whether the patient was ready for the transfer and adjust the transfer accordingly. This finding is similar to previous work that suggests that clinicians’ ability to be vigilant by collectively monitoring for potential patient deterioration and threats is an integral part of upholding safe collective practices in daily practice [39,40]. Interestingly, many of the care needs to be met are considered ‘basic elements of care’ including promoting hygiene, continence, hydration, and nutrition and managing pain; which are often overlooked in favor of other clinical competencies [41]. Further, the finding around the goal to transfer orthopedic surgical patients three days post-operatively may be too early for some patients and warrants further exploration to determine the extent and impact of this happening.

Our finding around ensuring that patient information needs are met is a key strategy for patient-centred care and has been associated with improved quality of care [42,43] and increased patient satisfaction [43-45]. Further there is growing interest in finding meaningful ways to engage patients and their caregivers in transitional care that promotes optimal health and minimizes the risk of errors [46-48]. Clearly, continued efforts on the part of leaders are necessary to enable clinicians to be vigilant around changing patient status and potential safety threats and identify and meet patient care needs during inter-organizational care transitions.

Our third theme around the need for accurate and complete documentation has been identified as key risk factors for negative outcomes associated with poor transitions [13,16,21,35]. Consistent with others [30,31,34], our study findings points to the need to centralize accessing patient information across health-care organizations including the use of information technology. There are increasingly calls to leverage information technology and create a centralized patient record which can be shared across health-care organizations [49]. A recent pilot study revealed promising signs using a web-based system to transmit critical patient information in real time to facilitate the care of patients referred to the emergency department by nursing homes [49]. Specifically, the study reported improved measures of information transfer and increased provider satisfaction. While electronic documentation is increasingly being deployed as a tool to improve the exchange of information, the adoption in handoff communication in care transitions remains limited [21].

### Study limitations

Our study has limitations that merit emphasis. First, the generalizability of study findings to other settings may be limited as the study participants were drawn from two sites only. Second, the data analyzed were from clinicians self-reporting their experiences with care transitions. Thus our findings may not reflect a comprehensive view of strategies from a clinician’s perspective to improve inter-organizational care transitions. These limitations are inherent in qualitative research and mitigating steps were taken with the sampling strategy that drew from a variety of clinicians (nurses, pharmacists, occupational therapists, physiotherapists, and dieticians) from both participating sites. Third, despite recruitment efforts, no physicians were interviewed for the study and thus their perspective on determining whether the patient is ready for transition and recommended strategies to improve care transitions is not included. Fourth, patients and their caregivers were not included in this dataset. While physicians, patients and caregivers are important groups, the multidisciplinary nature of patient care at both institutions ensured that many of the important aspects of transitions were identified.

To address these limitations, our study findings point to three key areas for future research. First, the exploration of the concept of mindlessness and strategies to mitigate the ways in which clinicians working in different settings may engage in actions that are counterproductive to collaboration and quality patient care are called for. This work would add to both the empirical base and growing theoretical explanations on how best to improve care transitions across the various health-care sectors and people’s homes. Second, future research should also focus on designing and testing out standard approaches to including a verbal report as part of the care transition process between health-care organizations. For example, a standard script that promotes enhanced professional hand-over of patients that includes a verbal exchange amongst clinicians at the sending and receiving organizations could be developed and tested. Third, further research is required to design and test the impact of centralized electronic documentation systems on information exchange during care transition points across sending and receiving health-care organizations. Fourth, given that the patient and their caregiver(s) are the most common factor across transitions of care, further exploration around addressing patients’ and the role of caregivers’ in improving care transitions is recommended.

## Conclusion

Our study points to the need for a collective and collaborative approach amongst clinicians to ensure the safe transition of patients across health-care organizations. Part of these efforts include having a written patient transfer record augmented with a verbal report where the receiving clinician can discuss with a clinician from the hospital the patient’s status on discharge and plan of care. Future research is required to design and test out interventions to optimize inter-organizational care transitions and feedback loops for complex medical patients.

## Competing interests

No competing interests (financial or non-financial) to declare.

## Authors’ contributions

The primary author (LJ) was the Principal Investigator 1) made substantial contributions to conception and design, acquisition of data, or analysis and interpretation of data; 2) drafted the article and revised it critically for important intellectual content; and 3) provided final approval of the version to be published. The other 3 authors (CB, JM, RL) contributed to the conception and design of the study, analysis of findings and contributed to the working drafts and final approval of the manuscript that was submitted.

## Pre-publication history

The pre-publication history for this paper can be accessed here:

http://www.biomedcentral.com/1472-6963/13/289/prepub
